# Studies of Phytochemicals, Antioxidant, and Antibacterial Activities of *Pinus gerardiana* and *Pinus roxburghii* Seed Extracts

**DOI:** 10.1155/2022/5938610

**Published:** 2022-05-31

**Authors:** Kanchan Bhardwaj, Rohit Sharma, Natália Cruz-Martins, Marian Valko, Navneet Kumar Upadhyay, Kamil Kuča, Prerna Bhardwaj

**Affiliations:** ^1^School of Biological and Environmental Sciences, Shoolini University of Biotechnology and Management Sciences, Solan 173229, India; ^2^Department of Rasashastra and Bhaishajya Kalpana, Faculty of Ayurveda, Institute of Medical Sciences, Banaras Hindu University, Varanasi, Uttar Pradesh 221005, India; ^3^Faculty of Medicine, University of Porto, 4200-319 Porto, Portugal; ^4^Institute for Research and Innovation in Health (i3S), University of Porto, 4200-135 Porto, Portugal; ^5^CESPU, Instituto de Investigação e Formação Avançada em Ciências e Tecnologias da Saúde, Rua Central de Gandra, 1317, 4585-116 Gandra PRD, Portugal; ^6^Faculty of Chemical and Food Technology, Slovak University of Technology, 81237 Bratislava, Slovakia; ^7^School of Pharmaceutical Sciences, Shoolini University of Biotechnology and Management Sciences, Solan 173229, India; ^8^Department of Chemistry, Faculty of Science, University of Hradec Kralove, 50003 Hradec Kralove, Czech Republic; ^9^Biomedical Research Center, University Hospital Hradec Kralove, Hradec Kralove, 50005 Hradec Kralove, Czech Republic

## Abstract

Pine seeds are considered as nonwood forest products (NWFP) with regularly increasing market's demand. They can be eaten in various ways such as roasted or raw. In addition, they are included in various traditional dishes like in cookies, sauces, candies, cakes, breads, and other bakery items and, moreover, for medicinal purposes. GC-MS study is performed to analyze the phytochemical compounds present in the seed extracts of *Pinus roxburghii* (Chir) and *Pinus gerardiana* (Chilgoza). In total, 25 compounds were identified each in Chir and Chilgoza. In Chir seeds, abundantly present compounds were 2,4-di-tert-butylphenol (16.6%), followed by ç-Terpinene (9.9%) and cyclohexanol, 4-ethenyl-4-methyl-3-(1-methylethenyl)-, (1à,3à,4á) (9.8%), whereas in Chilgoza seeds, the maximum amount of compound was 1-hexyl-1-nitrocyclohexane (17.3%), followed by phenol, 2,6-bis(1,1-dimethylethyl) (15.4%), and heptadecane, 2-methyl (8.4%). The total phenolic content of Chir seed sample was 1536 ± 4.35 (mg GAE/100 g), whereas in the Chilgoza seed extract was 642.66 ± 2.08 (mg GAE/100 g). The application of RP-HPLC-DAD system revealed that Chir and Chilgoza seeds have maximum quantity of catechin (15.77 ± 0.16 *μ*g/mg and 17.49 ± 0.32 *μ*g/mg, respectively). Both Chir and Chilgoza seed extracts exhibited significant antioxidant (radical scavenging) potential, through H_2_O_2_ (618.94 ± 21.45 *μ*g/mL and 575.16 ± 19.88 *μ*g/mL) and DPPH (552.60 ± 13.03 *μ*g/mL and 429.15 ± 3.80 *μ*g/mL) assays, respectively. Additionally, a well-known antibacterial potential was also found in both plants' dichloromethane extracts, with 64 to 256 *μ*g/mL of minimum inhibitory concentrations. As a whole, result shows the importance of both plants as a naturally occurring phytochemical source with significant antibacterial and antioxidant activity.

## 1. Introduction

Pine trees are one of the most common and widely grown species in the Himalayan region. Categorized under the *Pinus* genus and *Pinaceae* family, they are the largest conifer's families in the world and always remain evergreen woody conifer trees [1]. In the Himalayan region (HR), five species of pine are considered indigenous, distributed at different elevation such as *Pinus gerardiana* (Chilgoza), *Pinus wallichiana* (Kail), *Pinus roxburghii* (Chir), *Pinus merkusii* (Merkus), and *Pinus kesiya* syn. *Insularis* (khasi) as shown in Table 1 [2]. Among these, Chilgoza tree nuts have highly incomparable nutritious value therefore, commercialized for edible use and consumed in roasted form; these seeds are added as an ingredient in different dishes [3]. Whereas Chir trees are abundantly present and have ethno medicinal value, nuts are traditionally consumed in India and Pakistan [4, 5].

*P. roxburghii*, known as Chir pine trees, is generally 55 m tall and over 100 cm diameter breadth height (dbh). Tree bark is thick in dark reddish-brown while winter buds are brown, ovoid, small, and nonresinous. The tree leaves are needle-shaped in flabellate-triangular arranged in 3 per bundle and cylindrical in cross-section. Cones are pedunculate, short, ovoid, range in 10‐15 × 6‐9 cm, while seeds are small, about 8-12 mm in length with a long wing of 25 mm, and normally ripen in April [6].

*P. gerardiana* is traditionally called Chilgoza trees to a medium height (17 to 27 m) and 2-4 m in dbh. The branches are small and horizontal with glabrous bark and silver grey [7]. Leaves are needle-like shaped and dark green, arranged three per cluster. However, the male cones are longer than female ones, which are ovoid with hard woody scales. These seeds are in dark brown colored, pointed from top and cylindrical, and normally ripen in October [8].

Data available to date demonstrate that these trees are composed of different types of phenolic compounds, at varying amounts depending on the geographical origin, harvesting time and storage, with distinct biological activities being also reported [9]. In past two decades, massive studies were carried out on essential oils and extracts of several parts of pine trees. Studies generally focused on nutritional value, chemical composition, food supplements, and in drug formulation [10]. The natural bioactive compounds, including phenols, terpenes, flavonoids, alkaloids, and saponin obtained from different *Pinus* spp., have reported for their potency against several diseases, e.g., asthma, diabetes, neurodegenerative diseases, cancer, oxidative stress-related diseases, cardiovascular-related problems, liver and kidney disorders, and various pathogenic infections [2]. Among the phytochemicals present, these pine nuts usually contain inherent antioxidants that help in reducing the oxidation rate, namely, flavonoids, e.g., flavonols, and flavanones in various glycoside and aglycone form [11]. For instance various phenolic group constituents like as catechin, gallic acid and quercetin show antioxidant, antiallergic, antimicrobial, anti-inflammatory, UV protection, and anticancer activity [12, 13]. Ellagic acid has potency to inhibit the oxidation of low-density lipoprotein [14]. Vanillic acid is highly efficient to reduce oxidative stress and A*β*1-42-induced cognitive impairment, therefore very effective in Alzheimer's and other neuron-related disease [15].

Till now, studies have been carried out to assess the phytochemical composition and biological potential of Chilgoza seeds [3, 16]. In case of *P. roxburghii* except seed, the chemical composition and biological activities have been studied on its various parts, viz., needles and bark [4]. In this sense, the present study is aimed at evaluating the total phenolic content of *P. gerardiana* and *P. roxburghii* seed extracts and their bioactive compounds and at investigating the antioxidant and antibacterial roles. In addition, chemical composition and quantification of phenolic compounds were assessed by GC-MS and HPLC-DAD.

## 2. Materials and Methods

### 2.1. Chemical, Reagents, and Apparatus

2,2-Diphenyl-1-picrylhydrazyl (DPPH), Folin-Ciocalteu phenol reagent, L-ascorbic acid, di-sodium hydrogen phosphate de-hydrate, and sodium dihydrogen phosphate dehydrate were purchased from HiMedia Laboratories Pvt. Ltd. (India). HPLC grade water, sodium carbonate, and hydrogen peroxide were procured from Loba Chemie Pvt. Ltd. (India). Gallic acid, quercetin, ellagic acid, vanillic acid, catechin, epigallocatechin gallate (EGCG), and DCM were procured from Sigma-Aldrich (USA). LC–MS grade acetonitrile, methanol, and formic acid (of 98% purity) were procured from Fisher Chemicals (Hampton, NH, USA). Colistin was procured from HiMedia Laboratories Pvt. Ltd. (India). Labeled, CHROMAFIL Xtra poly ether sulfone (PES), 25 mm, and 0.20 *μ*m syringe filters were purchased from Macherey-Nagel (Düren, Germany). UV-vis spectrophotometer (Evolution 201) was procured from Thermo Fisher Scientific- Shanghai (China). ORBITEK shaker was purchased from Scigenics Biotech Pvt. Ltd. (India). Refrigerated centrifuge C-24 Plus was from REMI Sales and Engineering Ltd. (India). Analytical balance Aczet, CY2202 was purchased from Mettler-Toledo Pvt. Ltd. (India). Rotary vacuum evaporator-RE-52 was purchased from SONAR (India). Micropipette (20-200, 100-1000, and 0.5-10 *μ*L) of variable volume purchased from HiMedia Laboratories Pvt. Ltd. (India).

### 2.2. Preparation of Seed Extracts

At room temperature, de-shelled Chilgoza and Chir samples were grounded into powder with the help of commercial mixer grinder. Powdered seed samples of 50 g were added to 500 mL conical flask containing 250 mL dichloromethane (DCM) solvent each and kept in incubator cum shaker for 48 h at 37°C. Each sample was strained through Whatman no. 1 filter paper. After the extraction process, the liquid extracts were collected and then concentrated at 40°C by using a rotary vacuum evaporator. The prepared extracts were collected and stored at 4°C in refrigerator for further analysis [17].

### 2.3. Total Phenolic Content

The total phenolic content (TPC) of Chilgoza and Chir was determined by using Folin-Ciocalteau reagent [18]. Seed extracts (about 20 *μ*g) were separately taken and made volume up to 1 mL by adding distilled water. Then, Folin-phenol reagent (500 *μ*L) was added into that, and 2.5 mL sodium carbonate Na_2_CO_3_ (20%) was also added. It was mixed properly and kept away from light for 40 min for incubation and color development. Postincubation, the absorbance was taken at 725 nm. Gallic acid calibration curve was constructed, and linearity was found in 5-25 *μ*g/mL range. Seed extract TPC was stated in mg of gallic acid equivalent (mgGAE/100 g seed extracts) by using the standard curve.

### 2.4. GC-MS Study of the Seed Extracts

Each seed extract sample was diluted by adding DCM (1:10), and their fraction components were analyzed by GC-MS (TRIPLE QUAD GC-MS/MS) (Thermo Fisher, USA), equipped with an autosampler (TriPlusRSH) and DB 5 column (40 m × 0.15 mm i.d., film thickness of 0.15 *μ*m). For analysis of two different seed extracts, the following GC-MS operating conditions were followed with slightly modification as described by Al-Owaisi et al. [19]: initial temperature was kept at 80°C and time held for a min, thereafter with 10°C/min ramping rate reached up to 180°C by holding 2 min, and finally with same ramping increased to 260°C and was held for 15 minutes. Transfer line temperature, 250°C; carrier gas, He at constant flow rate 0.7 mL/min; split ratio, 71 : 4; 1 *μ*L of injection volume; component ionization, electron impact (70 eV) mode; EI source temperature, 230°C; m/z range, 45-450. The respected component relative concentrations were expressed as percent area on basis evolved in chromatograph. The identity of their components was done on the basis of visual interpretation and compared based on probability and literature search input as per NIST library (v. 2.2, 2014) with different types of compounds identified.

### 2.5. HPLC Analysis of the Seed Extracts

RP-HPLC system (Capcellpak) of Shimadzu (Kyoto, Japan) equipped well with a C-18 (2) column of phenomenex Luna (4.6 mm i.d. ×25 cm, 5 *μ*m) and a detector of diode array (DA) (SPD-M20A, Shimadzu, Japan) were taken for the phenolic compound quantification, including gallic acid, ellagic acid, vanillic acid, epicatechin, quercetin, catechin, and EGCG isomers [3]. Solvents used to run were as follows: solvent A: aqueous formic acid of 8% and solvent B: 10 : 90, *v*/*v* (acetonitrile/methanol). The flow rate was 0.9 mL/min. Injection volume was 20 *μ*L. The gradient was as given: 0 min, 20% B; 7 min, 35% B; 14 min, 45% B; 21 min, 65% B; 25 min, 85% B; and 32 min, 95% B. To monitor all the phenolic compounds, the DA detector wavelengths were fixed at 260, 280, or 320 nm. Standard solution preparation was done as described by Lee et al. [20]. From the stock, 0.01 mL was taken and diluted up to 1 mL diluents to make necessary dilutions and filtered through 0.22 *μ* PES membrane filters and injected in HPLC system.

### 2.6. Antioxidant Activities

#### 2.6.1. 2,2-Diphenyl-1-Picrylhydrazyl (DPPH) Free Radical Scavenging Assay

The free radical scavenging potential of two seeds' extract was evaluated following the method followed by Bhatti et al. [21] with slightly modifications. Stock solutions of both seeds' extracts (1 mg) were prepared in methanol (MeOH), and further different methanolic solution concentrations (20-640 *μ*g/mL) were prepared. From each concentration, 300 *μ*L was added to methanolic solution 2700 *μ*L of DPPH (4 mg/100 mL). The mixture solution was incubated in absence of light at 37°C room temperature for one hour. Free radical scavenging efficacy of extracts was based on the initial purple color disappearance. Absorbance of solution was taken at 517 nm. For positive control, ascorbic acid was used [21]. Scavenging capacity of DPPH was determined using the formula below given:

(1) $$\begin{array}{c} \text{DPPH radical scavenging activity }I\;\left(\%\right)=\frac{\left(\text{A blank}-\text{A sample}\right)}{\text{A blank}}\times 100, \end{array}$$

where the IC_50_ of DPPH radical was calculated from the line regression of the percentage of remaining DPPH radical against the sample concentration.

#### 2.6.2. Hydrogen Peroxide Scavenging Assay (H_2_O_2_)

The capacity of seed extract to scavenge H_2_O_2_ was estimated following the procedure of Bhatti et al. [21]. Briefly, 0.1 mL extract aliquots (20-640 *μ*g/mL) were added into an Eppendorf tubes to made volume up to 0.4 mL with addition of 50 mM (pH 7.4) phosphate buffer and (2 mM) H_2_O_2_ solution (0.6 mL). Mixture was properly vortexed and kept for 10 min, and then, absorbance was read at 230 nm. Ascorbic acid was used as positive control [21]. The extracts' ability to scavenge the H_2_O_2_ was evaluated by using the following equation:

(2) $$\begin{array}{c} {\text{H}}_{2}{\text{O}}_{2}\text{ scavenging activity percentage}=\left[\frac{\left(A_{0}-A_{1}\;\right)}{A_{0}}\right]\times 100, \end{array}$$

where *A*_0_ is the absorbance of blank and *A*_1_ is the absorbance of sample.

### 2.7. Antibacterial Screening by Minimum Inhibitory Concentration (MIC)

Gram-negative bacteria viz., *Salmonella typhimurium* MTCC 3224, Klebsiella pneumonia MTCC 109, and *Escherichia coli* MTCC 443 were used to study the antibacterial potential of both seeds' extracts, using the broth macrodilution technique [22]. DCM extracts were prepared in Mueller Hinton broth, and serial dilutions were obtained, ranging between 0.5 and 256 *μ*g/mL. Bacteria (1‐2 × 10^8^ CFU/mL) were transferred to test tubes and incubated at 37°C for 24 h. Minimum inhibitory concentrations (MIC) were determined, being considered as the lowest concentrations without visible turbidity. Colistin was used as a positive antibacterial control, while DCM was used as negative control [22].

### 2.8. Statistical Evaluation

Results obtained are expressed as mean value and standard deviation (*x* ± SD) with three times repeated trials for all experiments. The IC_50_ values were obtained by plotting inhibition-concentration curves by nonlinear regression analysis. Statistical analysis was performed using the Microsoft Excel.

## 3. Results and Discussion

### 3.1. Total Phenolic Content

The TPC of Chilgoza DCM seed extract was 642.66 ± 2.08 mg GAE/100 g, and TPC of Chir seed sample was 1536 ± 4.35 mg GAE/100 g. Hoon et al. reported that the maximum TPC in Chilgoza seeds was highest in water extract, followed by DCM, ethanol (EtOH), hexane (HEX), and ethyl acetate (EtOAc) as well as MeOH extracts [3]. Mahdhi et al. studied the high quantity of total phenols in *P. halepensis* methanolic-aqueous seed extracts 14.63 ± 0.05 mg/g gallic acid equivalent weight (GAE) [23]. Zulfqar et al. found higher TPC (77.2 ± 1.41 mg GAE/g) in methanolic extract of *P. gerardiana* dry nuts than in EtOAc extract (52.5 ± 2.9 mg GAE/g) [16]. Valero-Galván et al. found that *Pinus cembroides* grown in five states of Mexico presented different amounts of TPC in the methanol seed extract [24]. Bolling et al. report that TPC of pine nut was 68 mg GAE/100 mg; however, as per Phenol-Explorer database reports 58 mg GAE/100 mg [25]. Kadri et al. reported that Algerian pine species, viz., *Pinus pinea*, *Pinus halepensis*, *Pinus canariensis*, and *Pinus pinaster* seed extract, TPC differ within species [26]. Su et al. reported that *Pinus koraiensis* seed (PKS) ethanolic extract contained higher total phenolic content of 264 mg GAE/g [27].

### 3.2. GC-MS Analysis of the Seed Extracts

**Figure 1:**
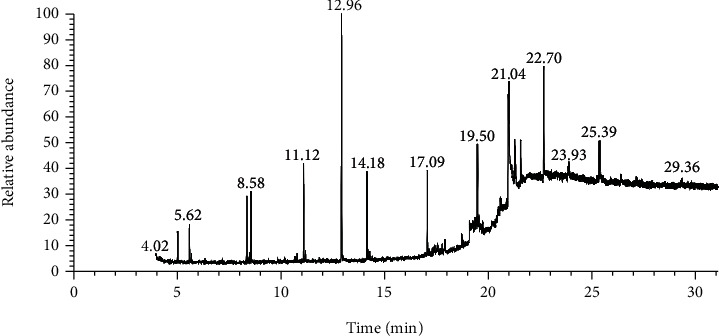
GC-MS profile of the DCM extract of Chilgoza with their retention time and peak assignment as in Table 2.

**Figure 2:**
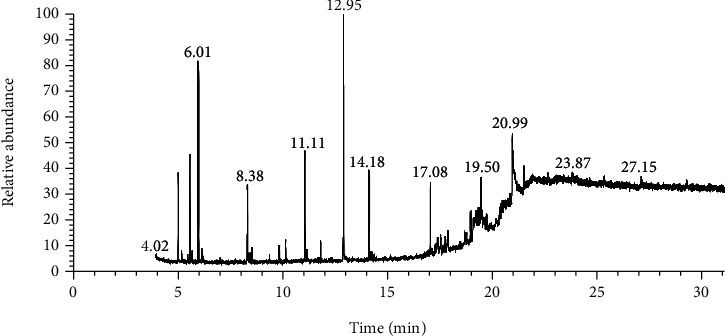
GC-MS profile of the DCM extract of Chir with their retention time and peak assignment as in Table 3.

Data obtained from the GC-MS analysis of DCM extracts of Chilgoza and Chir seeds revealed the presence of terpenoids, alcohols, alkenes, aromatic hydrocarbons, etc. (Figures 1 and 2). A total of 25 compounds were detected in Chilgoza and Chir, as shown in Tables 2 and 3. In Chilgoza seeds, the most abundant compound was 1-hexyl-1-nitrocyclohexane (17.3%), followed by phenol, 2,6-bis(1,1-dimethylethyl) (15.4%), and heptadecane, 2-methyl (8.4%), while in Chir seeds, 2,4-di-tert-butylphenol (16.6%), followed by ç-Terpinene (9.9%), cyclohexanol, 4-ethenyl-4-methyl-3-(1-methylethenyl)-, (1à,3à,4á) (9.8%) were the most commonly identified.

Kadri et al. reported *α*-pinene only in *P. pinaster*. Tables 4 and 5 display the biological activities of some compounds present in Chilgoza and Chir extracts, as reported in the various literatures [26]. However, GC-MS studies on pine nuts extracts were not updated in the literature; researchers emphasized on the essential oil analysis attained by extraction or emission from various tree parts, viz., branches, bark, cones, and needles [28].

### 3.3. HPLC Analysis of the Seed Extracts

Six compounds were detected by HPLC-DAD system based on available standards (Supplementary Table S1). The highest quantity was of catechin (10.49 ± 0.32 *μ*g/mg), followed by gallic acid (5.39 ± 0.39 *μ*g/mg), ellagic acid (5.21 ± 0.15 *μ*g/mg), vanillic acid (1.6 ± 0.12 *μ*g/mg), quercetin (0.84 ± 0.04 *μ*g/mg), and EGCG (0.15 ± 0.04 *μ*g/mg) were found in Chilgoza seeds. On the other hand, Chir seeds contained catechin (1.57 ± 0.16 *μ*g/mg), followed by ellagic acid (1.47 ± 0.06 *μ*g/mg), gallic acid (1.31 ± 0.08 *μ*g/mg), quercetin (1.28 ± 0.09 *μ*g/mg), and vanillic acid (0.27 ± 0.02 *μ*g/mg). EGCG was not detected in the Chir sample.

Hoon et al. also reported the highest quantity of catechin in Chilgoza DCM seed extract, but our data on EGCG contradict that obtained by this author [3]. Zulfqar et al. found that the maximum quantity of gallic acid (11.41 ppm) in methanolic extract of *P. gerardiana* dry nuts and in ethyl acetate extract quercetin was highest (165.33 ppm) [16]. Sadeghi et al. found maximum amounts of epicatechin (10.3 ± 0.18 *μ*g/mg), followed by catechin (10.1 ± 0.18 *μ*g/mg) in *Pinus eldarica* seeds grown in different regions of the Tehran province in Iran [39]. Mahdhi et al. compared eleven different phenolic compounds identified from *P. halepensis* methanolic-aqueous seed extract by convection-drying method and sun-drying method and reported that cirsiliol was chief flavonoid component, i.e., (0.761 and 1.916), than luteolin (0.589 and 1.760), followed by catechin (+) (0.569 and 0.888) and luteolin-7-O-glucoside (0.017 and 0.148) mg/100 g of dry weight, respectively [23].

### 3.4. Antioxidant Activities

The results of the antioxidant activity of Chilgoza and Chir seed extracts are shown in Table 6. Ascorbic acid showed stronger antioxidant activities in both DPPH (326.70 ± 9.64 *μ*g/mL) and H_2_O_2_ (375.73 ± 11.73 *μ*g/mL) assays as compared to both seed powder extracts tested. Comparing both extracts, the antioxidant potential of Chilgoza seed extracts in both DPPH and H_2_O_2_ assays was higher compared to that of Chir seed sample extract, which seems to be attributed to the presence of the high amount of antioxidant phytocompounds in Chilgoza samples as detected through the HPLC-DAD system, although the TPC in Chir seed samples was almost double than Chilgoza seeds.

However, earlier studies reported on pine nuts' phytochemical composition analyzes the presence of tocopherols, carotenoids, phytosterols, linoleic acids, and vitamin C, all of them revealing strong antioxidant potential, being their concentration higher than that in phenolic compounds [3, 9, 25]. The results obtained by DPPH assay to Chilgoza seeds DPPH were found accordingly with Hoon et al., who reported that an IC_50_ value of DCM seed extract was as good as compared to EtOAc, EtOH, HEX, and MeOH extracts. Moreover, Chilgoza water extract results were much better [3]. Zulfqar et al. reported that the percentage of DPPH inhibition of both MeOH and EtOAc extract of *P. gerardiana* dry nuts was 76.33 ± 2.51% and 73.67 ± 2.75% at concentration of 10 mg/mL and was statistically insignificant [16]. Valero-Galván et al. reported that methanol seed extract of *P. cembroides* grown in the five states of Mexico revealed different antioxidant activity assessed by DPPH assay [24]. *P. halepinsis* found in Palestine region displayed that methanolic extract by maceration and Soxhlet extraction method showed IC50 of 0.12 mg/mL and 0.43 mg/mL, respectively [10]. Mahdhi et al. studied maximum antioxidant activities from *P. halepensis* methanolic-aqueous seed extracts by using DPPH at concentration of 0.08 mg/mL [23]. Su et al. reported that *Pinus koraiensis* seed (PKS) ethanol extract displayed significant scavenging activity on 2,2-diphenyl-picrylhydrazyl (DPPH) (EC50, 0.023 ± 0.004 mg/mL) and significant suppressive effect on lipid peroxidation in liver as well as enhance the glutathione (GSH) and superoxide dismutase (SOD) antioxidant enzyme levels and reduce malondialdehyde (MDA) content in the brain and liver of rat [27]. Stem bark hydro alcoholic extract of *P. roxburghii*, *P. wallichiana*, and *P. gerardiana* showed significant IC_50_ value (*μ*g/mL) against DPPH at concentrations 97.54, 111.40, and 102.86, and ascorbic acid showed value at 18, respectively, and H_2_O_2_ (*μ*g/mL) showed IC_50_ value at 86.90, 84.18, and 81.83 while ascorbic acid showed 16.72, respectively [40].

### 3.5. Antibacterial Activity

The DCM extract of both Chilgoza and Chir seeds was found effective against Gram negative bacteria, viz., *S. typhimurium* MTCC. 3224, *K. pneumonia* MTCC. 109, and *E. coli* MTCC. 443. Results of seed extracts of Chilgoza and Chir antibacterial activity are shown in Table 7. Colistin was used as a positive control (MIC value was 8 *μ*g/mL).

Both Chilgoza and Chir seed antibacterial potentials were expected due to the occurrence of the antimicrobial compounds 3-carene, 2,4-di-tert-butylphenol, 1-hexyl-1-nitrocyclohexane, naphthalene, *α*-pinene, *γ*-terpinene, 1-undecanol, and 1-eicosanol, as reported in Tables 4 and 5. The possible target sites of phytocompounds in microbes are cell membrane, cell wall, and different enzymes. Salim et al. found that the antibacterial activities of *P. halepinsis* ethanolic seed extracts displayed good inhibition percentage against bacteria *Staphylococcus aureus*, *E. coli*, and *Shigella* at range of 0.02 g/mL [10]. Sharma et al. reported the antibacterial activity against *Pseudomonas aeruginosa*, *K. pneumonia*, and *E. coli* to the bark hydroalcoholic extract of three types of pine species, viz., *P. roxburghii* (Chir), *P. wallichiana* (kail), and *P. gerardiana* (Chilgoza) by well diffusion method, despite *P. wallichiana* displayed the most prominent antibacterial activity [40].

## 4. Conclusion and Future Perspectives

Secondary metabolites present in pine seed extracts are coated with excellent biological properties. In this study, the DCM seed extract of *P. gerardiana* and *P. roxburghii* revealed to be a rich source of molecules with interesting antioxidant and antimicrobial effects. The obtained results are positive and, if supported by *in vivo* studies, may be further proposed to be used for therapeutic purposes. In the near future, deeper studies on this field should be done, and other biological effects of such trees' extracts should also be carried out in experimental trials. Other studies should also be done to a better understanding of the impact of seed collection from different regions with altitudinal variation, in addition to correlation with climate, soil, and regional geographic data in chemical composition. Equally important will be to perform a combined analysis of protein, amino acid, minerals, and lipid profiles to reach a more clear understanding on the real potentialities of these less investigated trees.

However, the Chilgoza seeds are eaten in roasted form in several countries, but still Chilgoza and Chir seeds are not utilized in functional food development. Recently, our group has developed the cookies having Chilgoza and Chir seeds used in decorated form to enhance its nutritional value [41]. Still there is a need to develop functional/nutraceutical foods using Chilgoza and Chir seeds which ultimately gives new employment horizon to the hilly area people.

The limitation of the current study is the selection of extraction solvent. We believe that DCM solvent has not much compatibility with our seed samples; that was the reason we got antioxidants and antibacterial activity at higher concentration.

## Figures and Tables

**Table 1:** Geographical allocation of various pines species in HR [2].

Pine species	Habitat and distribution	Altitude (m)
*P. gerardiana*	Occur in drier rocky slope in some part of J&K and Kinnaur region of Himachal Pradesh	2000-3350
*P. wallichiana*	Found in the higher altitude of Himalayas drier region along with J&K up to Arunachal Pradesh north-eastern region of India	Till 2700
*P. roxburghii* Sarg.	Grows up and found in outer Himalayan valleys drier region of J&K to the Arunachal Pradesh	400-2300
*P. merkusii*	Found some regions in high moisture area of Indo-Myanmar border	1500
*P. kesiya* or *insularis*	Grows up in high moisture regions of Meghalaya north-eastern part of India	Up to 3000

**Table 2:** List of phytocompounds identified in Chilgoza by GC-MS.

Peak no.	Compound	RT	Area %	Mol. weight	Molecular formula	CAS. no.
1.	3-Carene	5.06	1.53	136	C_10_H_16_	13466-78-9
2.	2-Propyn-1-ol, acetate	5.62	2.27	98	C_5_H_6_O_2_	627-09-8
3.	1-Undecanol	8.38	3.14	172	C_11_H_24_O	112-42-5
4.	Naphthalene	8.58	3.81	128	C_10_H_8_	91-20-3
5.	8-Heptadecene	11.12	4.75	238	C_17_H_34_	2579-04-6
6.	Phenol, 2,6-bis(1,1-dimethylethyl)	12.96	15.42	206	C_14_H_22_O	128-39-2
7.	1-Hexadecanol	14.18	5.40	242	C_16_H_34_O	36653-82-4
8.	10-Heneicosene	17.09	4.77	294	C_21_H_42_	95008-11-0
9.	(2S,4R)-p-Mentha-[1(7),8]-diene 2-hydroperoxide	17.59	1.42	168	C_10_H_16_O_2_	NA
10.	2R-Acetoxymethyl-1,3,5-trimethyl-4c-(3-methyl-2-buten-1-yl)-1c-cyclohexanol	17.93	1.43	282	C_17_H_30_O_3_	NA
11.	Tetradecanoic acid, 10,13-dimethyl-, methyl ester	18.75	1.31	270	C_17_H_34_O_2_	267650-23-7
12.	Tetradecanoic acid, 12- methyl-, methyl ester, (S)	19.12	2.58	256	C_16_H_32_O_2_	62691-05-8
13.	Acetophenone, 2-[(p-nitrophenyl)imino]	19.21	1.60	254	C_14_H_10_N_2_O_3_	6394-60-1
14.	2-Methyl-3-(2,2-dimethylpropyl)-butadiene	19.41	1.20	138	C_10_H_18_	90822-87-0
15.	1-Nonadecene	19.50	6.08	266	C_19_H_38_	C19H38
16.	8-Isopropyl-5-methyl-5,6,7,8-tetrahydro-2,4-quinazolinedione	20.21	1.28	222	C_12_H_18_N_2_O_2_	63498-93-1
17.	4-tert-Octylphenol, TMS derivative	20.62	1.20	278	C_17_H_30_OSi	8721-87-6
18.	1-Hexyl-1-nitrocyclohexane	21.03	17.30	213	C_12_H_23_NO_2_	118252-09-8
19.	5,10-Pentadecadienoic acid, (E,E)-	21.29	2.34	238	C_15_H_26_O_2_	64275-68-9
20.	1,1,1,3,5,5,5-Heptamethyltrisiloxane	21.34	2.31	222	C_7_H_22_O_2_Si_3_	1873-88-7
21.	1-Hexyl-2-nitrocyclohexane	21.57	3.51	213	C_12_H_23_NO_2_	118252-04-3
22.	Heptadecane, 2-methyl	22.70	8.49	254	C_18_H_38_	1560-89-0
23.	Cyclotrisiloxane, hexamethyl	23.93	1.48	222	C_6_H_18_O_3_Si_3_	541-05-9
24.	4,4-Dipropylheptane	25.39	4.20	184	C_13_H_28_	17312-72-0
25.	1-propanone, 1-[5-ethyl-3-(5-nitro-2-furanyl)-1H-1,2,4-triazol-1-yl]	27.16	1.16	264	C_11_H_12_N_4_O_4_	35732-74-2

NA: not applicable.

**Table 3:** List of compounds identified in Chir by GC-MS.

Peak no.	Compound	RT	Area %	Mol. weight	Molecular formula	CAS no
1.	*α*-Pinene	5.06	4.59	136	C_10_H_16_	80-56-8
2.	3-Carene	5.62	6.22	136	C_10_H_16_	13466-78-9
3.	ç-Terpinene	6.01	9.99	136	C_10_H_16_	99-85-4
4.	1-Undecanol	8.38	4.11	172	C_11_H_24_O	112-42-5
5.	Oxirane, 2-(chloromethyl)-2-cyclopropyl	10.20	1.43	132	C_6_H_9_ClO	121505-35-9
6.	1-Hexadecanol	11.11	5.46	242	C_16_H_34_O	36653-82-4
7.	2,4-Di-tert-butylphenol	12.95	16.67	206	C_14_H_22_O	96-76-4
8.	10-Heneicosene	14.18	5.74	294	C_21_H_42_	95008-11-0
9.	1-Eicosanol	17.08	4.30	298	C_20_H_42_O	629-96-9
10.	5,10-Pentadecadienoic acid, (E,E)-	17.43	2.03	238	C_15_H_26_O_2_	64275-68-9
11.	2,6,10-Dodecatrien-1-ol, 3,7,11-trimethyl-9 (phenylsulfonyl)-, (E,E)	17.58	2.82	362	C_21_H_30_O_3_S	57683-67-7
12.	(2S,4R)-p-Mentha-[1(7),8]-diene 2-hydroperoxide	17.93	2.35	168	C_10_H_16_O_2_	NA
13.	2,6-Dimethyl-3,5,7-octatriene-2-ol	18.75	1.77	152	C_10_H_16_O	29414-56-0
14.	10,10-Dimethyl-2,6-dimethylenebicyclo[7.2.0]undecan-5á-ol	19.02	1.99	220	C_15_H_24_O	19431-80-2
15.	4-tert-Octylphenol, TMS derivative	19.14	2.01	278	C_17_H_30_OSi	78721-87-6
16.	2,3-Dimethylamphetamine	19.21	2.20	163	C_11_H_17_N	75659-60-8
17.	10-Pentadecen-5-yn-1-ol	19.29	1.43	222	C_15_H_26_O	64275-59-8
18.	1,1,1,3,5,5,5-Heptamethyltrisiloxane	19.40	1.48	222	C_7_H_22_O_2_Si_3_	1873-88-7
19.	1-Hexyl-2-nitrocyclohexane	19.50	4.56	213	C_12_H_23_NO_2_	118252-04-3
20.	1,2,4-Benzenetricarboxylic acid, 1,2-dimethyl ester	19.77	1.61	238	C_11_H_10_O_6_	54699-35-3
21.	3,4-Nonadien-6-yne, 5-ethyl-3-methyl	20.51	2.28	162	C_12_H_18_	61227-88-1
22.	4(3H)-Quinolinone, 3-hydroxy	20.57	1.75	161	C_9_H_7_NO_2_	55759-82-5
23.	Cyclohexanol, 4-ethenyl-4-methyl-3-(1-methylethenyl)-, (1à,3à,4á)	21.00	9.86	180	C_12_H_20_O	56298-45-4
24.	1-Hexyl-1-nitrocyclohexane 2-tert-butyl-3-(tert-butylimino)-4-phenyl	21.56	2.06	213	C_12_H_23_NO_2_	118252-09-8
25.	Thieno[2,3-b]pyridine, 5-ethyl-3-nitro	27.15	1.30	208	C_9_H_8_N_2_O_2_S	51043-51-7

NA: not applicable.

**Table 4:** Biological activities of Chilgoza seed compounds.

Compound name	Nature of compound	Structure	Biological activities	Ref
3-Carene	Monoterpenes	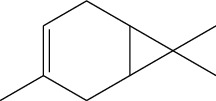	Antiacetylcholinesterase and antimicrobial	[29]
2,4-Di-tert-butylphenol or phenol, 2,6-bis(1,1-dimethylethyl)	Phenylpropanes	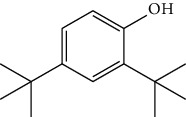	Antimicrobial, antioxidant, anti-inflammatory, cytotoxic, nematicidal, insecticidal, and allelopathic effect	[30]
1-Hexyl-1-nitrocyclohexane	Ketone	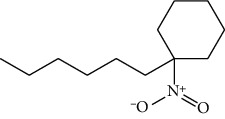	Antioxidant, antimicrobial, anti-inflammatory	[31]
1-Nonadecene	Alkene	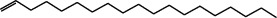	Antifungal, anticancer	[32]
Naphthalene	Aromatic hydrocarbon	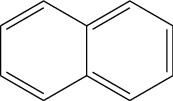	Anticancer, antiviral, antimicrobial, antidepressant, antineurodegenerative antidiabetic, anti-inflammatory, antitubercular, antihypertensive, antipsychotic, anticonvulsant	[33]

**Table 5:** Biological activities of Chir seed compounds.

Compound name	Nature of compound	Structure	Biological activities	Ref
*α*-Pinene	Monoterpene	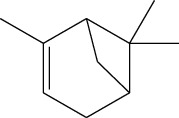	Antibiotic resistance modulation, antimicrobial, anti-*Leishmania*, antitumor, analgesic, antioxidant, anti-inflammatory, and antimalarial	[34]
ç-Terpinene	Monoterpene	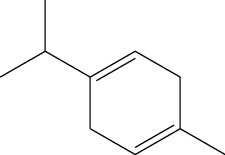	Antibacterial	[35]
9-Hexacosene	Alkene	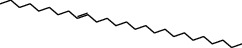	Analgesic and anti-inflammatory	[36]
1-Undecanol	Aliphatic alcohol	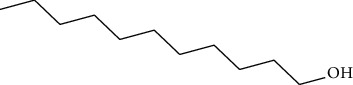	Bactericidal, larvicidal, and membrane-damaging activity	[37]
1-Eicosanol	Primary alcohols	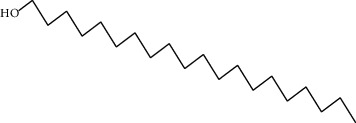	Antitumor and antibacterial activity	[38]

**Table 6:** Antioxidant activity of Chilgoza and Chir seed extracts on DPPH and H_2_O_2_ assays.

Plant name	Plant part extract	Mean IC_50_ value *μ*g/mL ± standard deviation	
		DPPH	H_2_O_2_
*Pinus gerardiana* (Chilgoza)	Seed	429.15 ± 3.80	575.16 ± 19.88
*Pinus roxburghii* (Chir)	Seed	552.60 ± 13.03	618.94 ± 21.45
Ascorbic acid (control)		DPPH (326.70 ± 9.64 *μ*g/mL)	H_2_O_2_ (375.73 ± 11.73 *μ*g/mL)

**Table 7:** Antibacterial activities of Chilgoza and Chir seed extracts.

Plant name	Plant part extract/MIC *μ*g/mL	Bacteria
*Pinus gerardiana* (Chilgoza)	Seed/128	*Salmonella typhimurium* MTCC 3224
*Pinus gerardiana* (Chilgoza)	Seed/128	*Klebsiella pneumonia* MTCC 109
*Pinus gerardiana* (Chilgoza)	Seed/256	*Escherichia coli* MTCC 443
Plant name	Plant part extract/MIC *μ*g/mL	Bacteria
*Pinus roxburghii* (Chir)	Seed/128	*Salmonella typhimurium* MTCC 3224
*Pinus roxburghii* (Chir)	Seed/64	*Klebsiella pneumonia* MTCC 109
*Pinus roxburghii* (Chir)	Seed/128	*Escherichia coli* MTCC 443

## Data Availability

The data used to support the findings of this study are available from the corresponding authors from request.

## Supplementary Materials

Supplementary Materials Table S1: retention time of different flavonoids.
