# Supplementary material for: Studies of Phytochemicals, Antioxidant, and Antibacterial Activities of Pinus gerardiana and Pinus roxburghii Seed Extracts
Source: Biomed Res Int. 2022 May 31;2022:5938610. doi: 10.1155/2022/5938610 (PMC9173889; doi:10.1155/2022/5938610)
Supplement: Supplementary Materials — Table S1: retention time of different flavonoids. [file 5938610.f1.doc]

Table S1: Retention time of different flavanoids.

| S.No. | Name of Standard | Retention Time (min.) | Area |
| --- | --- | --- | --- |
| 1. | Gallic acid (100µg) | 3.692 | 148754 |
| 2. | Ellagic acid (10ug) | 5.590 | 10982 |
| 3. | Catechin(100µg) | 6.584 | 26601 |
| 4. | EGCG (100µg) | 7.279 | 83830 |
| 5. | Vanillic acid (100µg) | 11.597 | 568992 |
| 6. | Qurecetin (100µg) | 22.548 | 435984 |
